# Isolation, Characterization and Antitumor Effect on DU145 Cells of a Main Polysaccharide in Pollen of Chinese Wolfberry

**DOI:** 10.3390/molecules23102430

**Published:** 2018-09-21

**Authors:** Fei Chen, Linwu Ran, Jia Mi, Yamei Yan, Lu Lu, Bo Jin, Xiaoying Li, Youlong Cao

**Affiliations:** 1 College of Public Health and Management, Ningxia Medical University, Yinchuan 750004, China; chenfei150113@163.com; 2 Laboratory Animal Center, Ningxia Medical University, Yinchuan 750004, China; 3 Institute of Wolfberry Engineering Technology, Ningxia Academy of Agriculture and Forestry Sciences, Yinchuan 750002, China; lorna0102@126.com (J.M.); lulubing8901@163.com (L.L.); 15509585650@163.com (B.J.); lxy_2002_79@163.com (X.L.); youlongchk@163.com (Y.C.)

**Keywords:** Chinese wolfberry, pollen polysaccharides, purification, antitumor activity

## Abstract

Modern studies have shown that pollen has a certain role in the treatment of prostate-related diseases. In the present study, pollen polysaccharides from Chinese wolfberry (WPPs) were extracted by hot-water extraction and ethanol precipitation, further purified by chromatography on a DEAE-cellulose column and Sephadex G-100 column. Homogeneous polysaccharide CF1 of WPPS was obtained, the molecular weight of which was estimated to be 1540.10 ± 48.78 kDa by HPGPC-ELSD. HPLC with PMP derivatization analysis indicated that the monosaccharide compositions of CF1 were mannose, glucuronic acid, galacturonic acid, xylose, galactose, arabinose, and trehalose, in a molar ratio of 0.68:0.59:0.27:0.24:0.22:0.67:0.08. The antitumor effects of CF1 upon MTT, Tunel assay and flow cytometry assay were investigated in vitro. The results showed that CF1 exhibited a dose-dependent antiproliferative effect, with an IC50 value of 374.11 μg/mL against DU145 prostate cancer cells. Tunel assay and flow cytometry assay showed that the antitumor activity of CF1 was related to apoptosis in vitro. The present study suggested that the CF1 of WPPs might be a potential source of antitumor functional food or agent.

## 1. Introduction

Prostate cancer is an epithelial malignancy that occurs in the prostate and is a malignant tumor with a high incidence in the male genitourinary system. Although treatments such as surgery, radiochemotherapy or androgen ablation have improved the survival rate of patients, a cure remains elusive, with resistance developing over time [1]. Therefore, finding a low-toxic and effective anti-cancer drug from natural compounds has become a hot topic in cancer treatment. Pollen has been used as a “perfect health food” for many centuries due to its abundance of nutrimental constituents and bioactive compounds [2]. It contains a variety of natural products such as polysaccharides, phytosterols, flavone, terpenoids and saponins. In the 1990s, studies reported that pollen water extract Cernitin T-60 could inhibit the growth of prostate cancer cells DU-145 and LNCap [3]. Modern research has also shown that pollen possesses therapeutic effects [4]. Pollen of flowers is a natural product with a long history of use in China and Europe as a well-tolerated treatment for prostate-related diseases such as benign prostatic hyperplasia and prostatitis [5]. Polysaccharides are one of the main active ingredients of pollen. Much research has indicated that polysaccharides from pollen significantly inhibit tumor growth and enhance immunomodulating activity [6,7,8,9,10]. Chinese wolfberry is a traditional Chinese medicine used for both medicine and food. Modern research shows that it has many functions, such as improving the immune function, anti-oxidation, anti-aging, antitumor and liver protection [11,12]. At present, much research is dedicated to the study of the polysaccharides from wolfberries, like antitumor activities on tumor cells such as those in the respiratory, reproductive, urinary and digestive systems [13,14,15,16]. However, little is known about the antitumor properties of polysaccharides from wolfberry pollen. Therefore, in this study, we isolated polysaccharides from wolfberry pollen (WPPs) and analyzed their monosaccharide compositions and molecular weights. Then, the in vitro antitumor activities of isolated polysaccharides were evaluated by 3-(4,5-dimethylthiazol-2-yl)-2,5-diphenyltetr-razolium bromide (MTT), Tunel assay and flow cytometry assay in DU145 prostate cancer cells. To the best of our knowledge, this is the first study to investigate the antitumor activity of pollen polysaccharides from the Chinese wolfberry.

## 2. Results

### 2.1. Extraction, Isolation and Purification of WPPs

**Figure 1:**
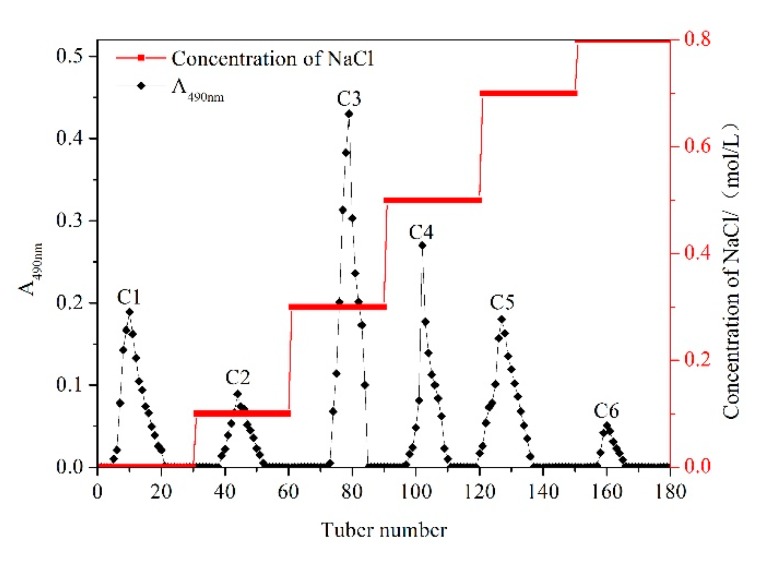
Gradient elution profile of polysaccharides extracted from Chinese wolfberry pollen by DEAE cellulose-52 chromatography.

Hot water extraction and ethanol precipitation methods were carried out to extract crude WPPs. Subsequently, purification of the crude WPPs was performed by DEAE cellulose-52 column, and they were eluted with gradients of 0, 0.1, 0.3, 0.5, 0.7, and 0.8 mol/L NaCl solutions. The fractions of the isolated WPPs were mainly concentrated in the elution solution. As shown in Figure 1, six components were separated and obtained from WPPs. Among these, the content of C3 was the highest, so the C3 component was selected and further purified by the Sephadex G-100 column.

### 2.2. Further Purification by Sephadex G-100 Column

**Figure 2:**
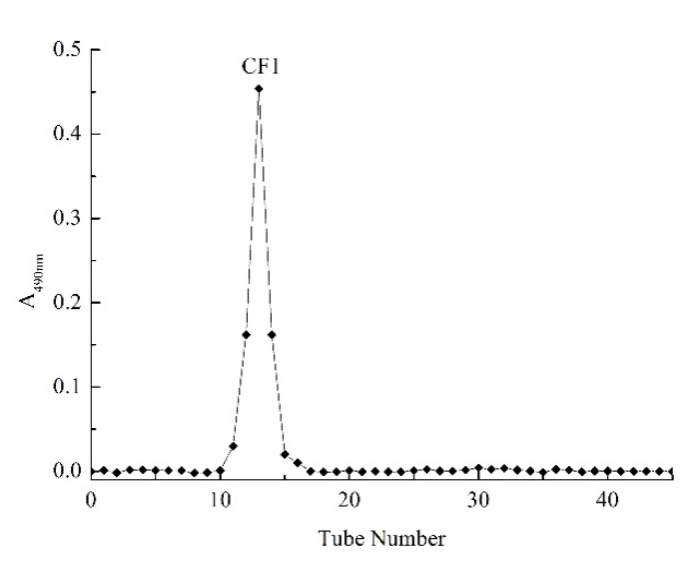
Elution profile of polysaccharide CF1 by Sephadex G-100 column chromatography.

The separated C3 fraction of the WPPs was further purified using a Sephadex G-100 column. The results are shown in Figure 2. One component further separated and purified by the Sephadex G-100 column was a single eluting symmetrical peak, indicating that the obtained polysaccharide was a relatively homogeneous polysaccharide. After dialysis and lyophilization of the collected solution of this component, a pure polysaccharide CF1 was obtained.

### 2.3. Analysis of Molecular Weight and Monosaccharide Composition of CF1 of WPPs

**Figure 3:**
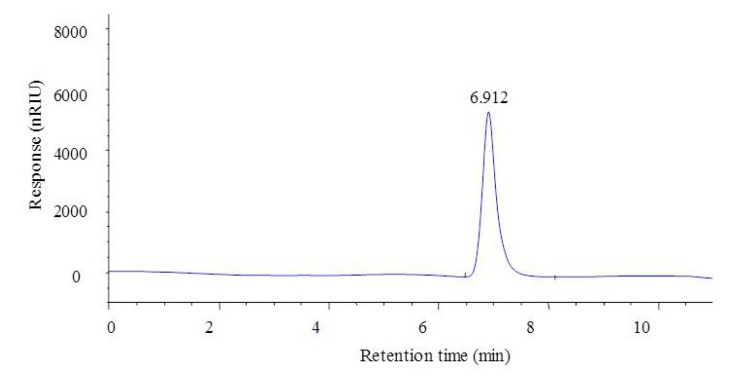
HPLC spectra of CF1 purified from the C3 fraction of the WPPs.

**Figure 4:**
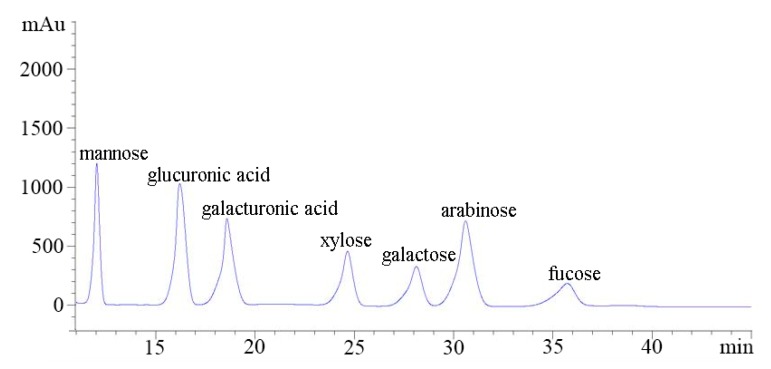
HPLC spectra of monosaccharide compositions of CF1.

The molecular weight of the CF1 of WPPs was determined using High-Performance Gel permeation Chromatography-Evaporative Light Scattering Detector (HPGPC-ELSD). The molecular weight of CF1 is shown in Figure 3; the elution peak of the CF1 isolated and purified from the polysaccharide C3 was single and symmetrical. It was shown that the CF1 isolated and purified by the two columns was separated. The retention time of the CF1 component was 6.961 min. The molecular weight (MW) calculated on the basis of the standard pullulan molecular weight curve was 1540.10 ± 48.78 kDa. The monosaccharide compositions of CF1 were analyzed by PMP derivatization, and consisted of mannose, glucuronic acid, galacturonic acid, xylose, galactose, arabinose, and trehalose in a molar ratio of 0.68:0.59:0.27:0.24:0.22:0.67:0.08 (Figure 4), indicating that it was a kind of heteropolysaccharide.

### 2.4. Effects of CF1 of WPPs on the Proliferation of DU145 Cells

**Figure 5:**
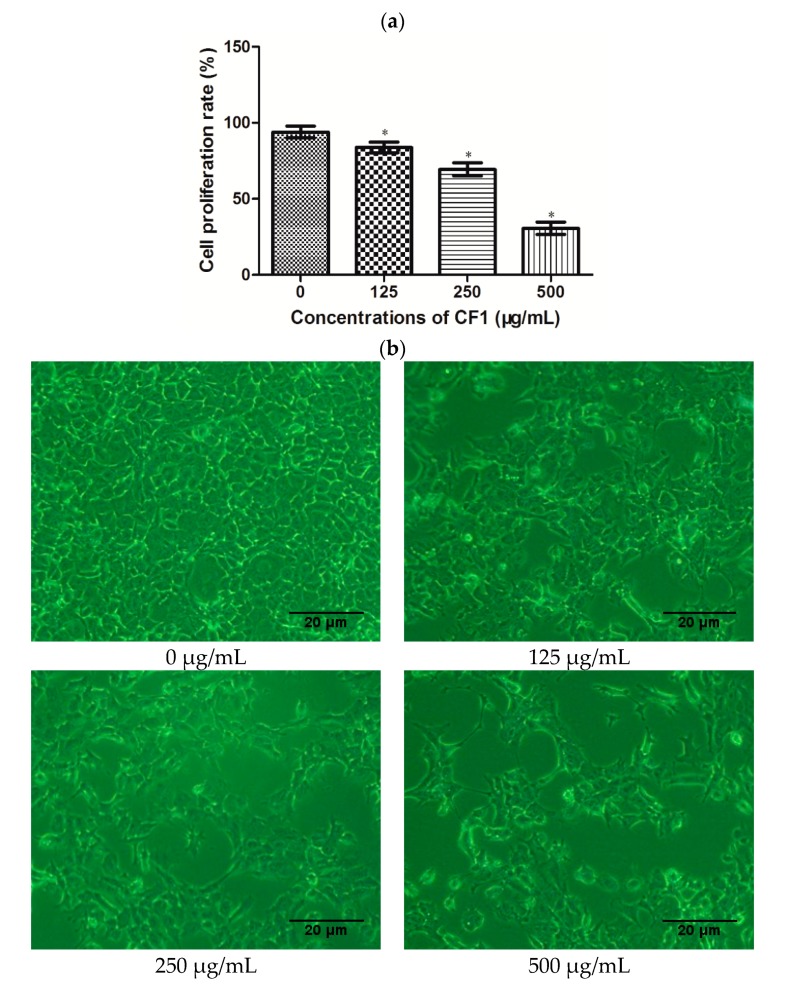
Effects of the CF1 of WPPs on the proliferation of DU145 cells. (**a**) DU145 cells were treated with 0, 125, 250, 500 μg/mL CF1 of WPPs in Dulbecco’s Modified Eagle’s Medium (DMEM) containing 10% FBS for 24 h, and the proliferation rate was measured by an MTT assay. The data are presented as the mean ± SD (*n* = 3), * indicates a significant difference, (*p* < 0.05). (**b**) Morphological changes of DU145 cells treated with different concentrations of CF1 for 24 h.

In this study, we used an MTT assay to measure the proliferation rate of DU145 cells. As shown in Figure 5a, there was a significant decrease in the proliferation of cells with increasing doses of CF1 (*p* < 0.05). The relative proliferation rates of the DU145 cells were 85.3%, 72.0% and 33.6%, respectively, after treatment with CF1 (125, 250, and 500 μg/mL) for 24 h. The CF1 of WPPs inhibited the growth of DU145 cells, with an IC50 value of 374.11 μg/mL. As shown in Figure 5b, the morphology of cells was observed under an inverted microscope. In the control group, cells well adhered to the wall and grew firmly, showing an elliptical shape and cell density. In contrast, cells treated with CF1 at the concentrations of 125, 250, and 500 μg/mL became round, could not be affixed to the walls, and floated in the medium. The number of cells treated with CF1 also decreased compared to the control group. The morphological changes of DU145 cells were consistent with results of MTT assay. These results indicated that the CF1 of WPPs significantly inhibited the proliferation of DU145 cells in a dose-dependent manner.

### 2.5. Effects of the CF1 of WPPs on the Apoptosis Induction of DU145 Cells

**Figure 6:**
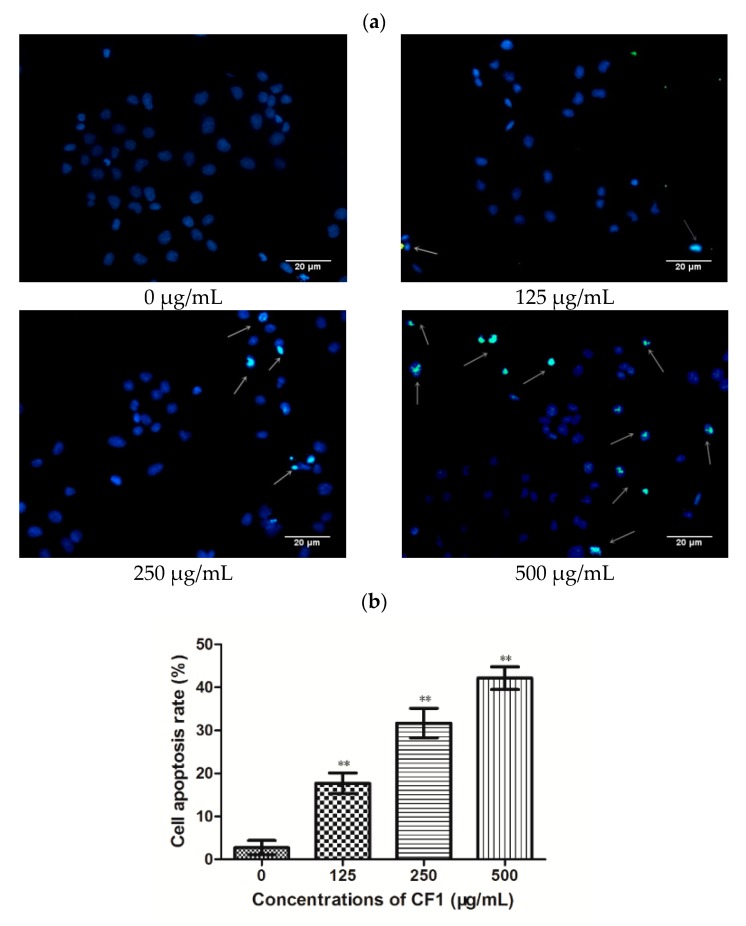

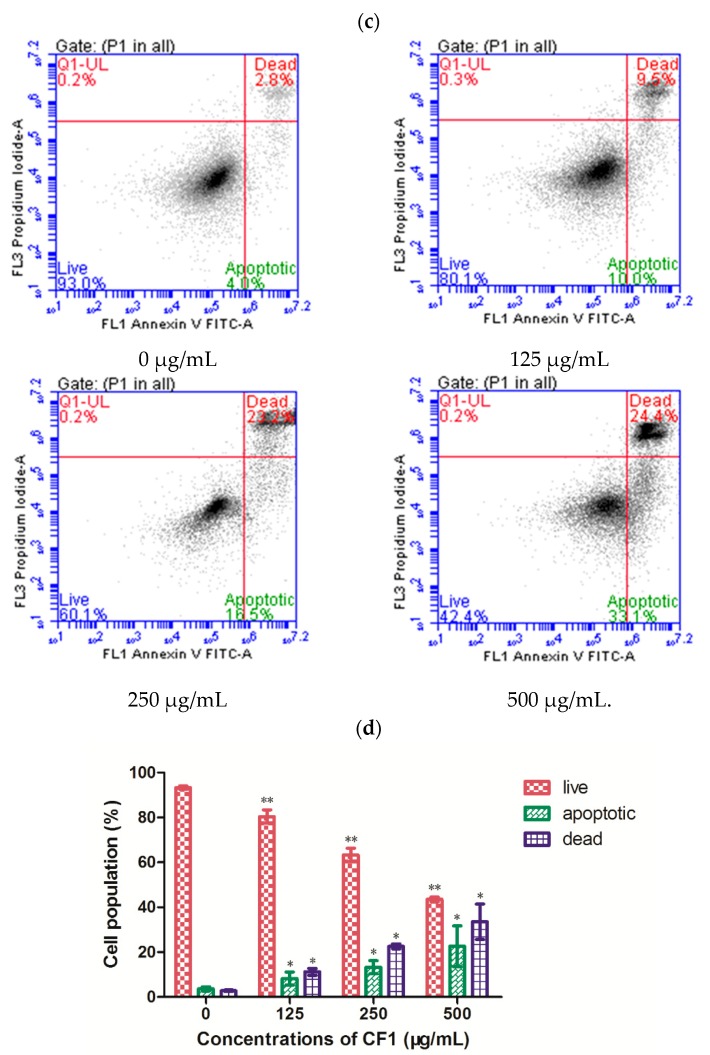
Induction of apoptosis in DU145 cells by CF1 of WPPs. (**a**) Apoptosis in DU145 cells detected by TUNEL assay. DU145 cells were treated with CF1 at concentrations of 0, 125, 250, 500 μg/mL for 24 h and were then subjected to Tunel assay and imaged by microscopy. The condensed and fragmented nuclei are indicated with arrows. (**b**) Cell apoptosis rate is expressed as the mean ± SD (*n* = 3). * *p* < 0.05; ** *p* < 0.01 compared with the control. (**c**) Apoptosis in DU145 cells detected by flow cytometry. (**d**) The data are presented as the mean ± SD (*n* = 3). * *p* < 0.05, ** *p* < 0.01 compared with the control.

Apoptosis is considered a major cause of cell growth inhibition. To investigate whether the CF1 of WPPs’ suppression of DU145 cells proliferation is attributable to apoptosis, we used a Tunel assay to observe apoptosis in DU145 cells. In the event of apoptosis, endogenous DNA endonuclease activation causes DNA fragmentation and 3′-OH exposure. Exposure of 3′-OH can be labeled deoxynucleotide, which can be displayed in situ by fluorescence. The nucleus of the apoptotic cells that were stained with Tunel showed green fluorescence, and DAPI-stained nuclei showed blue fluorescence. Therefore, the apoptotic cells were blue-green in the merged fluorescence of the two-color fluorescence. A certain number of apoptotic cells were seen in each CF1 treatment group, and as the concentration increased, the number of apoptotic cells increased (Figure 6b). These results of the Tunel assay suggested that the CF1 of WPPs could induce the apoptosis of DU145 cells.

Furthermore, we also used flow cytometry to measure distribution of cells in an early and the later stages of the apoptosis. As seen in Figure 6c,d, the CF1 of WPPs significantly induced apoptosis in DU145 cells in a dose-dependent manner. The induction of apoptosis rates in DU145 cells were 10.0%, 16.5% and 33.1% (*p <* 0.05), respectively, after treatment with CF1 (125, 250, and 500 μg/mL) for 24 h. The results of the live and dead assay of DU145 cells were consistent with the results of the apoptosis assay. These results suggested that the CF1 of WPPs inhibited the growth of DU145 prostate cancer cells through induction of apoptosis.

## 3. Discussion

Pollen not only contains various nutrient chemical components, but also contains complex bioactive substances such as polysaccharides, flavonoids, saponins, and phytosterols. Modern studies have shown that pollen still has a certain role in the treatment of diseases, especially in antitumor, anti-oxidation, lipid-lowering, anti-inflammatory [4,17,18]. Polysaccharides are one of the main active components of pollen. Polysaccharides have been reported to possess a broad spectrum of biological activities, particularly antitumor [19,20,21,22] and immunomodulating activities [23,24,25,26].

Previous studies have indicated that different extraction methods have effects on the structure and bioactivity of polysaccharides. A study by D. Cör et al. revealed that the antioxidant activity of polysaccharide-protein complexes attained by ultrasound-assisted extraction was generally higher than with the conventional hot-water method [27]. Zhu et al. confirmed that hot-water extraction also had a higher extraction efficiency [28]. Thus, it would be interesting to pursue a further study of extraction methods with regard to the yield and antitumor activity of pollen polysaccharides from Chinese wolfberry. The polysaccharide is composed of more than 10 monosaccharide residues polymerized by glycosidic bonds. Different kinds of polysaccharides contain different types of monosaccharides, different degrees of polymerization, different properties of glycosidic bonds, and its different MWs will lead to differences in the physical properties and biological activity of the polysaccharides. Generally, higher MW molecular weight is associated with greater antitumor activity [29]. Moreover, polysaccharides with higher MW had a more complex structure and composition [30]. In this study, the molecular weight of CF1 component was estimated to be 1540.10 ± 48.78 kDa. In addition, several observations have suggested that the monosaccharide compositions of the polysaccharides could affect their antitumor activity [31,32]. Moreover, polysaccharides with β-glucan such as (1→3)-linked-β-d-glucose backbone chain exert a relatively higher anti-proliferation activity [33]. Our results of monosaccharide compositions of CF1 were similar to those findings.

Human prostate cancer cell lines are composed of hormone-sensitive and -insensitive cells. LNCaP is a human androgen-dependent prostatic cancer cell line. DU145 and PC-3 cells are hormone insensitive. In this study, human prostate cancer cell line DU145 was chosen to evaluate the proliferation effects of CF1 of WPPs in vitro using the MTT method. The results indicated that CF1 of WPPs possessed growth inhibition effects against DU145 cells, and the proliferation rate exhibited a dose-dependent behavior. These results are consistent with previous research in following by treatment with paeonol [34] and fucoidan [35]. The cytotoxic effects of wolfberry polysaccharides have previously been reported on several tumor cell lines, including PC-3 and DU-145 (prostate cancer cells) [36], SW480 and Caco-2 (colon cancer cells) [13], and QGY7703 (hepatoma cells) [37]. Nevertheless, there have been no reports on the antiproliferative properties of WPPs.

Apoptosis is a kind of programmed cell death. Once this regulatory mechanism is broken, it will lead to the occurrence of disease, tumors and even death. Therefore, inducing apoptosis and inhibiting cancer progression has become a new perspective on the treatment of cancer. To determine whether DU145 cells treated with CF1 of WPPs underwent apoptosis, Tunel assay and flow cytometry were used to observe apoptotic cells. Our results showed that the CF1 of WPPs also significantly induced apoptosis in DU145 cells in a dose-dependent manner in vitro. Furthermore, we confirmed that the antitumor effects of the CF1 of WPPs were related to induction of apoptosis and death of DU145 cells by flow cytometry assay. Induction of apoptosis is one important mechanism by which an antitumor agent acts [38]. Some previous research has demonstrated that polysaccharides induce the apoptosis of cancer cells by promoting the expression levels of cleaved poly ADP-ribose polymerase (PARP) and caspase-3 [39,40,41]. Therefore, it is worthwhile to have a further study of the mechanism for the effects of CF1 on DU145 prostate cancer cell apoptosis.

## 4. Materials and Methods

### 4.1. Materials and Reagents

The wolfberry pollen was collected from the Ningxia Academy of Agriculture and Forestry Sciences (Yinchuan, Ningxia, China). Cellulose DEAE-52, Sephadex G-100 columns and monosaccharide standards (mannose, glucuronic acid, galacturonic acid, xylose, galactose, arabinose, and trehalose) were purchased from Sigma Chemical Co., Ltd. (St. Louis, MO, USA). HPLC-grade acetonitrile was purchased from TEDIA Co., Inc. (Fairfield, IA, USA). 1-phenyl-3-methyl-5-pyrazolone (PMP) high-glucose DMEM medium and fetal bovine serum were purchased from Gibco (Carlsbad, CA, USA). Penicillin-streptomycin double antibody and 0.25% trypsin were purchased from Solarbio Science & Technology Co., Ltd. (Beijing, China). 3-(4,5-dimethylthiazol-2-yl)-2,5-diphenyltetrazolium bromide (MTT) was purchased from KeyGEN BioTech Co., Ltd. Nanjing, China). In situ cell death detection kit and POD were purchased from Roche Diagnostics (Indianapolis, IN, USA) and DAPI was obtained from ZSGB-BIO Biological Co., Ltd. (Beijing, China). Annexin V-FITC cell apoptosis detection kit was purchased from Best Bio Biological Co., Ltd. (Shanghai, China).

### 4.2. Extraction of Crude WPPs

The preparation of wolfberry pollen was carried out according the reported method [42] with some modifications. The procedure of extraction was as follows:

Firstly, 200 g of wolfberry pollen was crushed and extracted using five-fold volume of petroleum ether at room temperature for 5 h, and the organic layer was removed by chromatography paper filtration, then the residue was extracted by five-fold volume of deionized water for 3 times, each time for 2 h. The extraction temperature was 45 °C The combined aqueous extracts were filtered by chromatography paper and concentrated by a four-fold volume of anhydrous ethanol at 4 °C overnight. The precipitates were collected by centrifugation at 4000 rpm for 15 min, dissolved in 5 mL deionized water and dialyzed for 48 h. At last, the extract was decompressed by rotary evaporator (Shanghai Yarong Biochemical Instrument Factory, Shanghai, China) at temperature below 45 °C and then freeze-dried for 24 h to obtain crude WPPs.

### 4.3. Purification of Crude WPPs by DEAE Cellulose-52 Column

Crude WPPs (100 mg) were dissolved in deionized water (20 mL), and this solution was slowly added to the well-balanced DEAE cellulose-52 column using a dropper along the column wall. Gradient elution was sequentially performed with 0, 0.1, 0.3, 0.5, 0.7 and 0.8 mol/L NaCl solutions at a flow rate of 1 mL/min, and 20 tubes were collected per gradient for 10 min each. The phenol-sulfate acid method was used to detect the polysaccharide content, and the elution curve was plotted according to the content. Firstly, different volume (0, 0.2, 0.4, 0.6, 0.8, 1 mL) glucose solution were took into the tubes and added water to 2 mL; secondly, 1 mL of 6% phenol solution and 5 mL of concentrated sulfuric acid were added to those tubes, reacted for 20 min, and then measured the absorbance at 490 nm. The sample with the highest polysaccharide content was selected, collected and concentrated by rotary evaporator (below 45 °C). The salt was removed by dialysis and then lyophilized.

### 4.4. Further Purification by Sephadex G-100 Column

The polysaccharide fraction obtained in the previous step was mixed with 10 mL of a 5 mg/mL solution in deionized water and slowly added to the equilibrated Sephadex G-100 column. Elution was performed with deionized water at a rate of 0.3 mL/min, and elution fractions were automatically collected, 10 mL per tube. The Phenol-sulfate acid method was used to detect the absorbance of the collected samples and to draw the elution gradient curve. The sample with the highest polysaccharide content was selected and collected by a rotary evaporator, concentrated, dialyzed, desalted and lyophilized to obtain pure WPPs.

### 4.5. Analysis of Monosaccharide Composition of CF1 of WPPs

The monosaccharide composition was determined by HPLC by the described method of pre-column derivatization with PMP [43]. The specific methods were as follows: Firstly, the monosaccharide standards were mixed into a mixed aqueous solution (the concentration of each monosaccharide was 5 mg/L). 50 μL of the mixed standard monosaccharide solution was pipetted into 50 μL 0.6 M NaOH solution and mixed well. Then the mixture (100 μL) was labeled with 1-phenyl-3-methyl-5-pyrazolone (PMP) by adding 100 μL of 0.5 M methanol solution of PMP. The following reaction took place in a 75 °C constant-temperature blast oven for 110 min, at which point it was then removed and cooled down. 0.3 M hydrochloric acid (100 μL) was added for neutralization and then evaporated. Next, the extraction was performed three times with chloroform. The aqueous layer was filtered through a 0.22 μm membrane and analyzed by HPLC.

To the polysaccharide sample solution (100 μL, 2 mg/mL) was added 4 M trifluoroacetic acid (TFA, 100 μL) to hydrolyze at 110 °C for 2 h. After hydrolysis, methanol solution (200 μL) was added to remove excess TFA, and then evaporated at reduced pressure. This procedure was repeated again to remove the TFA. The residue was dissolved in 100 μL of 0.3 M NaOH solution. Then 100 μL of 0.5 M methanol solution of PMP was added to the mixed solution and the reaction was carried out at 70 °C for 100 min. After cooling, the solution was neutralized, extracted, and filtered using a 0.22 μm membrane in the same manner as that used for the mixed standard monosaccharide solution.

### 4.6. Determination of the MolecularWeight of CF1 of WPPs

High-performance gel permeation chromatography (HPGPC, TSK G4000PWXL column 7.8 × 300 mm, Tosoh Crop, Tokyo, Japan)—Evaporative Light Scattering Detector (ELSD) was used to measure the molecular weight of the polysaccharide fraction after two stepwise purifications of DEAE cellulose-52 and Sephadex G-100. In addition, distilled water was used as the mobile phase, the flow rate was 0.8 mL/min, and the temperature of the column was 35 °C. At the same time, the pullulan polysaccharide standards whose molecular weight range was close to that of the samples were selected (the molecular weight was 20, 100, 200, 400, 800, 1600 kDa) as a standard curve, according to the standard curve to obtain a linear regression equation as follows: y = −0.5423x + 9.734.435 (R^2^ = 0.9973); y is the logarithm of the molecular weight of the polysaccharide; x is the retention time of the pollen polysaccharide (min). Based on the retention time of the pollen polysaccharide on the column and the standard pullulan molecular weight curve of the column, the molecular weight of WPPs was calculated.

### 4.7. Antitumor Activities of the CF1 of WPPs In Vitro

#### 4.7.1. Cell Culture

Human androgen-independent DU145 cell were bought from Stem Cell Bank, Chinese Academy of Sciences (Shanghai, China). Cells were cultured in DMEM, supplemented with 10% fetal bovine serum and 1% penicillin-streptomycin at 37 °C in a humidified 5% CO_2_ incubator (Thermo Fisher Scientific, Shanghai, China). These cells were maintained in the appropriate medium and passaged when cells cover 90% of the bottom of the culture flask.

#### 4.7.2. Cell Proliferation Assay

Cell proliferation was measured by an MTT assay. Briefly, exponentially growing cells were made into cell suspensions at a concentration of 1 × 10^5^/mL, then 100 μL of the cells were seeded into a 96-well plate (1 × 10^4^/well), an additional 100 μL of fresh DMEM medium was added. After the 96-well plate was maintained at 37 °C with 5% CO_2_ in a humidified CO_2_ incubator for 24 h, different concentrations (0, 125, 250, 500 μg/mL) of CF1 of WPPs were then added into a 96-well plate and to the control group (0 μg/mL) was also added an equal volume of medium and incubated for 24 h. Following incubation, MTT solution (50 μL, 1mg/mL) was added and further incubated for another 4 h. The medium containing MTT in the well was discarded, and then 150 μL of dimethylsulfoxide (DMSO) was added and homogenized thoroughly at room temperature for 10 min to fully dissolve the crystals. The absorbance of the cells was determined in a full-wavelength micoplate reader (at 550 nm). All experiments were performed at least three times and were calculated using average results. The inhibition rate was calculated by the following formula:

 Proliferation rate = (ODtreatedODcontrol)×100% 

#### 4.7.3. Tunel Assay

The apoptosis was assessed by Tunel assay. Exponentially growing cells were collected, and the density adjusted to 1 × 10^5^/mL, inoculated in 1 mL per well in a 6-well plate, until the cells adhered to the culture flask. Then, 1 mL medium containing different concentrations of CF1 of WPPs was added; the control group was added to the same volume of fresh medium, and incubated for 24 h. After the treatment, cells were fixed with 4% paraformaldehyde for 30 min at room temperature, and then washed with PBS three times. After washing, they were incubated with 3% H_2_O_2_-methanol for 10 min at room temperature then washed with PBS 3 times. Fixed cells were then permeabilized with 0.1% Triton X-100 for 10 min on ice. After washing, the Tunel reaction mixture was prepared, the treatment groups were mixed with enzyme solution (50 μL) and fluorescein-labeled dUTP solution (450 μL); however, only 50 μL fluorescein-labeled dUTP solution was added to the control group and incubated in a wet box for 1 h at 37 °C. This was followed by washing with PBS and DAPI was added for 5 min. Finally, the apoptotic cells were viewed and counted by a fluorescent microscope (Olympus BX43, Tokyo, Japan).

#### 4.7.4. Flow Cytometry Assay

The DU-145 cells were treated with different concentrations of CF1 of WPPs and the cell apoptotic ratio was determined by Annexin V-FITC and PI staining followed by analysis with flow cytometry. Early and late apoptotic changes in different cells were determined using an Annexin V-FITC/PI Apoptosis Detection Kit (BestBio, Shanghai, China) following the manufacturer’s instructions. Cells (1 × 10^6^/mL) were collected and washed twice with precooled PBS. The samples were resuspended in 400 μL 1 × Annexin V; To the cell suspension was added 5 μL Annexin V-FITC staining solution, gently mixed and incubated for 15 min at 2 °C to 8 °C in the dark, then 10 μL PI staining solution was added and gently mixed at 2 °C to 8 °C in the dark, incubated for 5 min and immediately tested with a FACS Calibur flow cytometer (BD Biosciences, San Joes, CA, USA).

### 4.8. Statistical Analysis

All experiments were repeated at least three times, statistical analysis was carried out using SPSS 17.0 statistical software. All values were presented as the mean ± SD, multi-group mean comparison was analyzed by one-way variance, and pairwise comparison was performed by LSD test. Significance difference was set at *p* < 0.05.

## 5. Conclusions

In summary, a polysaccharide CF1 with molecular weights of 1540.10 ± 48.78 kDa, was successfully isolated from wolfberry pollen through DEAE-52 cellulose and Sephadex G-100 chromatography. Monosaccharide analysis revealed that the CF1 of WBPPs was composed of mannose, glucuronic acid, galacturonic acid, xylose, galactose, arabinose, and trehalosein in a molar ratio of 0.68:0.59:0.27:0.24:0.22:0.67:0.08. The biological activity of the CF1 of WPPs exhibited a dose-dependent antitumor effect on the DU145 prostate cancer cell. The antitumor activity of CF1 was related to apoptosis in vitro. However, the mechanism of how the CF1 of WPPs induces apoptosis is unclear; we need further research and exploration. In a word, CF1 of WPPS should be considered as a potential source of antitumor functional food or agent.
